# Variation in Phenolic Compounds Content and Antioxidant Activity of Different Plant Organs from *Rumex crispus* L. and *Rumex obtusifolius* L. at Different Growth Stages

**DOI:** 10.3390/antiox8070237

**Published:** 2019-07-23

**Authors:** Pavel Feduraev, Galina Chupakhina, Pavel Maslennikov, Natalia Tacenko, Liubov Skrypnik

**Affiliations:** Institute of Living Systems, Immanuel Kant Baltic Federal University, Kaliningrad 236000, Russia

**Keywords:** *Rumex* L., polyphenols, catechins, antioxidant activity, DPPH, ABTS, FRAP

## Abstract

The study investigated the accumulation of phenolic compounds and the antioxidant activity of extracts of various parts of *R. crispus* and *R. obtusifolius*, collected at the flowering stage and the fruiting stage. Half of the collected plants were divided into root, stem, leaves, and reproductive organs (inflorescence). The other half was used to study the vertical distribution of biologically active components and antioxidants throughout the plant. The samples were analyzed for total catechins content, total proanthocyanidins content, total phenolic content, and total antioxidant activity (1,1-diphenyl-2-picrylhydrazyl (DPPH), 2,2’azinobis(3)ethylbenzthiazoline-6-sulfonic acid (ABTS), and ferric reducing antioxidant power (FRAP) assays). All analyses were performed in four replicates. In general, a similar trend was observed in the distribution of phenolic compounds in the studied species. The maximum content of these secondary metabolites was noted in the reproductive organs, both in the flowering and fruiting period. Stems were characterized by a minimum content of the studied classes of substances. The antioxidant activity of the sorrels studied parts can be arranged in the following order: the generative part (flowers, seeds) > leaves > root > stem (for flowering and fruiting stages). It was found that parts of the root closer to the stem differed in higher activity.

## 1. Introduction

To date, the search for bioactive compounds from new natural sources remains a relevant topic for analysis. We hold that addressing this question will improve the quality of food, the general standard of living, and public health. In this regard, phenolic compounds are bioactive components of particular interest. Many studies have shown that plant-derived polyphenols have anti-aging, anti-inflammatory, and antiproliferative properties, and proved their effectiveness in reducing the risk of developing cardiovascular deceases, cancer, and diabetes [1,2,3,4,5]. A study by Transparency Market Research, a global market research team, predicted a boom on the polyphenol market due to growing demand and the size of the market with an annual growth rate of 6.1% [6]. 

Phenolic compounds participate in redox reactions and in the neutralization processes of active forms of oxygen, which is known to have positive effects on human health [7,8,9]. Unclarified plant extracts containing the whole variety of phenolic compounds, and the individual groups and compounds of this class have been found to possess high antioxidant properties [10,11,12,13,14,15,16]. More recently, the use of antioxidants for the treatment of various diseases has become increasingly controversial [17,18,19]. In particular, the use of synthetic antioxidants gives rise to serious doubts [20,21]. However, previous studies have found that introduction of vegetable products having antioxidant properties into the daily diet significantly reduces the risk of developing cardiovascular, neurodegenerative, and oncological deceases [22,23,24]. Therefore, a more extensive study and application of plants belonging to the *Polygonaceae* family is of increasing importance, as they synthesize a great variety of biologically active secondary metabolites, including such phenolic compounds as anthraquinones; stilbenes; catechins; flavonoids; and their glycosides, leucoanthocyanins, and phenolic acids [25]. 

The *Polygonaceae* family consists of plants of the *Rumex* L. genus, which contains about 200 species widely distributed throughout the world. For a long time, plants of this genus have been used as food products, medicines in folk medicine, and dyes [26]. Some species of the *Rumex* L. genus are cultivated, for example, *R. acetosa* and *R. vesicarius*, whereas others are invasive weeds (for example, *R. obtusifolius* and *R. crispus*) [27,28,29]. The latter has a fast growth rate, high efficiency of biomass accumulation, and resistance to adverse environmental factors.

*Rumex crispus* L., known as the curly dock or yellow dock, is common in most of Europe, North Africa, Turkey, Northern Iran, Central and East Asia, and North America [30]. The roots of this plant have been used in folk medicine as a tonic and laxative, as an astringent for bleeding, as well as for rheumatism and some skin diseases. The fruits (seeds) are used for the treatment of dysentery. Young leaves of *R. crispus* are edible and are often consumed as vegetable, especially in the spring [28,31]. Phytochemical analysis showed the presence of anthracene derivatives (chrysophanol; physcion; emodin; and their glycosides, rhein, nepodin, nepodin-1-O-β-D-glycoside, 1.5-dihydroxyanthraquinone, oxymethyloanthraquinone, and glucofranguline), trioxybenzoic acid, catechins, quercetin, kaempferol, and their glycosides in the *R. crispus* plants [32,33,34]. 

*Rumex obtusifolius* L. (broad-leaved dock) is an invasive weed, which is widespread in Western and Central Europe, in the Scandinavian countries, on the Balkan Peninsula, in the Mediterranean, in Asia Minor, and in Iran [30]. In folk medicine, a decoction made from fruits, stems, leaves, and roots of the plant is commonly used as an astringent, laxative, and tonic, as well as for the treatment of ulcers, blisters, burns, and diabetes [35,36]. Furthermore, a series of phenolic compounds such as anthracene derivatives (emodin, chrysophanol, physcion, aloe-emodin, rhein, nepodin, frangulin-emodin, frangulin-emodin-glycoside, and nepodin-8-glycoside), flavonoids, and procyanidins have been identified in plants of *R. obtusifolius* [32,33,34]. 

Biosynthesis of secondary metabolites, including phenolic compounds, is a dynamic process, mostly dependent on numerous factors associated with the plant itself and with the environment. Among the first group of factors, the most important ones are the type of plant and the phase of its ontogenetic development. The accumulation and concentration of polyphenolic compounds in different parts and organs of plants can also vary greatly, which is closely related to the function of these compounds in plants’ lifecycle and the growth phase [32,37,38,39,40]. 

In this regard, the present study was undertaken in an effort to investigate the accumulation of phenolic compounds and the antioxidant activity of various parts and organs of plants *R. crispus* and *R. obtusifolius* during the flowering and fruiting stages.

## 2. Materials and Methods

### 2.1. Plant Material

Samples of plants *R. crispus* and *R. obtusifolium* were taken at the stages of flowering (according BBCH scale stage 64) and fruiting (according BBCH scale stage 81) from June to August 2017 in the areas of natural growth of these species. The plant material was collected from the village Lesistoe, geographically located in the natural park “Vishtynetsky” (specially protected natural area), belonging to the Nesterovsky district of the Kaliningrad region of Russia. Plants grew at a height above sea level from 13 to 25–30 m. The meadow plant community selected for the collection of plant material showed a deficient degree of anthropogenic load because it was not agricultural land, it was located far away from the transport routes; and there was no production in the adjacent territories. We identified the plant species using the “Illustrated determinant of plants of Central Russia” and compared the collected material with samples of *R. crispus* and *R. obtusifolium* stored in the KLGU Herbarium. Ph.D. Volodina A. determined the herbaria specimens [41]. It is worth noting that these species have a high tendency to hybridize; for this reason, we collected each species at a considerable distance from each other, but within the boundaries of a specific phytocenosis. Therefore, all the studied plants can be considered as being in homogenous ecological and climatic conditions. 

**Figure 1:**
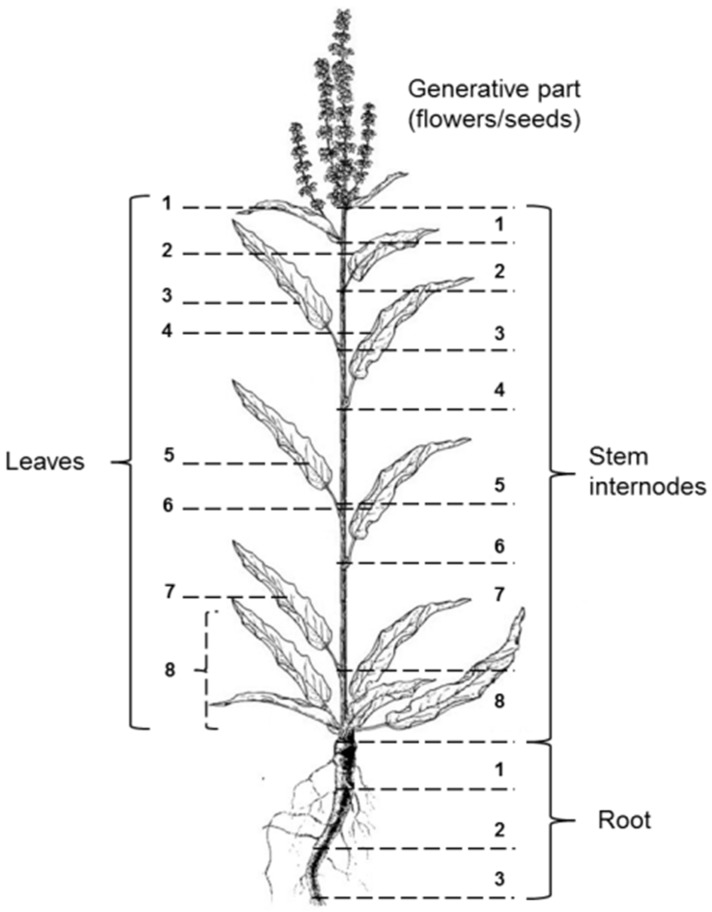
Scheme of the analysis of the vertical distribution of phytocomponents in sorrel plants.

A total of eight plants of each species was collected in each phase of ontogenetic development. All selected plants were in good condition without any visible signs of mechanical and infectious damage. Half of the collected plants were divided into root, stem, leaves, and generative part. The other half was used to study the vertical distribution of biologically active components and antioxidants throughout the plant. To this end, the stem was divided into eight internodes that were analysed separately. The leaves from each node also constituted a separate sample. The root was divided into three parts, having dimensions equal to the size of the internodes of the stem. Figure 1 presents the scheme of separation and numbering of the studied parts of sorrel plants. Preliminary sample preparation took place under laboratory conditions: the material was washed and dried to air-dry, and then to a dehydrated state at 60 °C. The absolutely dry material was ground to the size of particles passing through a sieve with a hole diameter of 2 mm. We then prepared average test samples, from which weighed samples necessary for performing a specific analysis were taken.

### 2.2. Plant Analysis

Plant extract preparation: 0.1–0.2 g plant material was homogenized with 10 mL of 96% ethanol solution, centrifuged at 4500 g for 30 min. The supernatant was used for analysis.

### 2.3. Total Catechins Content (TCC)

The vanillin method was used for determination of catechins [42]. In pre-prepared tubes with 4 mL of vanillin reagent (2.5 mL of a 5% alcohol solution of vanillin + 47.5 mL of concentrated HCl) was poured 1 mL of plant extract, starting with a blank solution (1 mL ethanol). The contents of each tube were mixed and transferred to the cuvettes. The absorbance was measured 5 min later after adding the extract to the vanillin reagent at a wavelength of 520 nm (UV-3600, Shimadzu, Japan). The results were expressed as mg of catechin equivalent per g of dry weight (mg CE g^−1^).

### 2.4. Total Proanthocyanidins (PAs) Content 

Proanthocyanidins (PAs) content was measured by buthanol–HCl assay [43]. Briefly, 0.5 mL of the ethanolic extract was added to 3.0 mL of butanol–HCl reagent (butanol/HCl, 95:5; *v/v*) and 0.1 mL 2% ferric reagent (2% ferric ammonium sulfate in 2 M HCl), after which test tubes were vortexed and put into a boiling water bath for 60 min. After cooling, absorbances were recorded at 550 nm (UV-3600, Shimadzu, Japan) against the blank, containing 0.5 mL of solvent instead of the extract. The results were expressed as mg of cyanidin equivalent per g of dry weight (mg CyE g^−1^).

### 2.5. Total Phenolic Content (TPC)

Total phenolics content was determined by the Folin–Ciocalteu method [44]. Briefly, 100 μL of gallic acid standard or plant extract was mixed with 300 μL 0.2 M Folin–Ciocalteu reagent in a tube and incubated for 10 min at room temperature in darkness. Next, 6 mL of 6.75% sodium carbonate (Na_2_CO_3_) solution was added to each tube, and the tubes were incubated for 30 min at room temperature in darkness. The optical density of the above solution was determined at 765 nm (UV-3600, Shimadzu, Japan). TPC was expressed as mg gallic acid equivalent per gram dry weight (mg GAE g^−1^).

### 2.6. Total Antioxidant Activity (AOA)

The total antioxidant activity was measured using DPPH (1,1-diphenyl-2-picrylhydrazyl) radical, ABTS^+^ (2,2’azinobis(3)ethylbenzthiazoline-6-sulfonic acid) radical, and FRAP (ferric reducing antioxidant power) assays. Each extract was mixed with 2.85 mL freshly prepared 0.1 mM solution of DPPH in ethanol. The sample was incubated for 30 min at room temperature in darkness. The reduction of absorbance at 515 nm (UV-3600, Shimadzu, Japan) was measured spectrophotometrically [45]. ABTS and FRAP assays were performed as described by Taneva et al. [46]. ABTS radical was generated by mixing aliquot parts of a water solution of 7.0 mM (ABTS) and 2.45 mM potassium persulfate. For the assay, 2.85 mL of this ABTS^+^ solution was mixed with 0.15 mL of obtained extracts. After 15 min at 37 °C in darkness, the absorbance was measured at 734 nm (UV-3600, Shimadzu, Japan) against ethanol. 

The FRAP reagent was freshly prepared by mixing 10 parts 0.3 M acetate buffer (pH 3.6), 1 part 10 mM 2,4,6- tripyridyl-triazine (TPTZ) in 40 mM HCl, and 1 part 20 mM FeCl_3_×6H_2_O in dH_2_O. The reaction was started by mixing 3.0 mL FRAP reagent with 0.1 mL of investigated extract. The reaction time was 10 min at 37 °C in darkness, and the absorbance was measured at 593 nm (UV-3600, Shimadzu, Japan) against blank prepared with ethanol. All results from the determination of antioxidant capacity were expressed as μmol Trolox equivalents per gram dry weight (μmol TE g^−1^).

### 2.7. Statistical Analysis

All analyses were performed in four replicates. One-way analysis (ANOVA) was performed using the SigmaPlot 12.3 (Systat Software GmbH, Erkrath, Germany). Because three-factorial ANOVA detected significant interactions between all factors (Table 1), one-factorial ANOVA was conducted for each factor (species, plant part, growth stage) separately. Before ANOVA, the data were checked for normality and the homogeneity of variance. Statistical comparisons were performed using the Tukey’s multiple comparison test. Differences were considered significant at *p* ≤ 0.05. A correlation analysis based on Pearson’s chi-squared test was conducted. The graphs with means and SD were performed using OriginPro 9 (OriginLab Corporation, Northampton, MA, USA).

## 3. Results

### 3.1. Total Catechins Content

**Figure 2:**
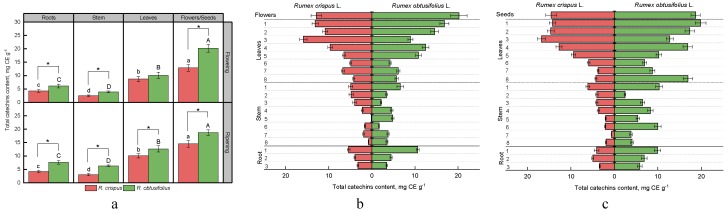
The content of catechins in different parts of plants *R. crispus* and *R. obtusifolius* at the stages of flowering and fruiting (**a**), and vertical distribution of catechins at flowering stage (**b**) and at fruiting stage (**c**). Different lower case letters indicate significant differences among plant parts of *R. crispus*; upper case letters indicate significant differences among plant parts of *R. obtusifolius* (*p* ≤ 0.05); and asterisk * indicates significant differences among sorrel species (*p* ≤ 0.05) based on post hoc Tukey’s tests separately for each growth stage.

Our work studied the total content of catechins in the roots, stem, leaves, and the generative part of plants *R. crispus* и *R. obtusifolius* at the flowering and fruiting stages (Figure 2a). 

All parts of the *R. obtusifolius* plants were proven to show a higher level of these compounds.

Depending on the stage of growth, the difference in the number of phytocomponents between the species was 1.4–1.8 times in the roots, 1.6–2.1 times in the stem, 1.2–1.3 times in the leaves, and 1.6–1.3 times in flowers. The maximum content of catechins for both species was noted in the generative part. In flowers, their level was 12.9 ± 1.2 and 20.2 ± 1.5 mg CE g^−1^; and in seeds, it was 14.5 ± 1.2 mg CE g^−1^ and 18.7 ± 1.1 mg CE g^−1^, for *R. crispus* and *R. obtusifolius*, respectively. 

The smallest accumulation of catechins was observed in the stems of both species, which was five times lower compared with the flowers at the flowering stage and 2.3–4.8 times lower than that of the seeds at the fruiting stage. During the transition from flowering to fruiting, the content of catechins in the stem, leaves, and the generative part of *R. crispus* plants increased, while in the roots, their level practically did not change. For *R. obtusifolius*, the content of this class of compounds also increased, except for the generative part, indicating a slight decrease in the content of catechins.

A study of the accumulation of catechins in three parts of the root, eight internodes of the stem, and leaves on the nodes showed the variability of the distribution of the compounds throughout the plant. Both species contained a significantly higher level of catechins in the first part of the root, which is located more closely to the ground, at the flowering stage (Figure 2b). A particularly pronounced difference between the root parts studied was noticeable for *R. obtusifolius* plants, where the content of catechins was comparable to their level in some leaf samples and reached 10.6 ± 0.3 mg CE g^−1^, which was 2.4–3.1 times more than in the rest of the root. The stems of both species indicated a general trend towards increasing content of catechins from the lower parts to the high (younger) ones. 

A similar trend was found for the studied leaf samples. Younger leaves, located closer to the generative part of plants, contained higher levels of catechins. It is worth reporting that the maximum of catechins for *R. crispus* was detected in the leaves of the third node from the top of the stem. For *R. obtusifolius,* a significantly higher content of catechins was determined in the leaves of the first and second nodes. 

At the fruiting stage, there were subtle variations in the distribution of catechins in the plant (Figure 2c). Analysis of different parts of the *R. crispus* root showed an almost homogenous distribution of catechins (3.4–4.9 mg CE g^−1^), whereas in *R. obtusifolius*, a higher content was noted in the first part (9.9 ± 0.7 mg CE g^−1^) as opposed to the lower parts (5.8–6.9 mg CE g^−1^). In the stalk and leaves of *R. crispus*, there is a tendency to increase the content of catechins from the lower parts to the upper ones at the fruiting stage, while the stalk of *R. obtusifolius* contains a high level of catechins at the sixth, fourth, third, and first internodes. In addition, high content of catechins was noted in the root leaves of this species (eighth node from the top of the stem), as well as in the leaves of the fourth, second, and first nodes located closer to the generative part. 

### 3.2. Total Proanthocyanidins Content (PAs)

**Figure 3:**
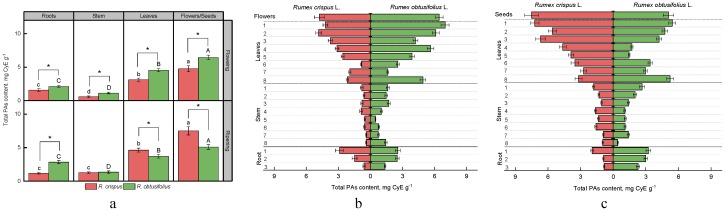
The content of proanthocyanidins in different parts of plants *R. crispus* and *R. obtusifolius* at flowering and fruiting stages (**a**), and vertical distribution of proanthocyanidins at flowering stage (**b**) and at fruiting stage (**c**). Different lower case letters indicate significant differences among plant parts of *R. crispus*; upper case letters indicate significant differences among plant parts of *R. obtusifolius* (*p* ≤ 0.05); asterisk * indicates significant differences among sorrel species (*p* ≤ 0.05) based on post hoc Tukey’s tests separately for each growth stage.

Figure 3a presents the results of the study of the total content of proanthocyanidins (condensed tannins) in different parts of the plants *R. crispus* and *R. obtusifolius*. Plants of the species *R. obtusifolius* indicated higher values at the flowering stage. 

In the *R. obtusifolius* roots, the level of proanthocyanidins was 1.4 times higher; in the stem, it was 2.0 times higher; in leaves, it was 1.5 times higher; and in flowers, it was 1.4 times higher compared with these parts of *R. crispus*. The *R. obtusifolius* roots contained more proanthocyanidins at the fruiting stage. Approximately the same content of these compounds was determined in the stems of both species. However, a higher level of proanthocyanides was revealed in the leaves and seeds of *R. crispus*, with the latter showing the most significant difference. The generative part of *R. crispus* contained 1.5 times more proanthocyanidins compared with *R. obtusifolius*. 

We observed the maximum accumulation of proanthocyanidins in the generative part (4.8–6.4 mg CyE g^−1^) of both species studied, and the minimum accumulation in the stem (0.6–1.1 mg CyE g^−1^) during the flowering stage. At the fruiting stage, there was no significant difference in the level of proanthocyanidins in the root and stem of *R. crispus*, while the seeds of both species contained higher amounts of these components (5.1 and 7.5 mg CyE g^−1^ in *R. obtusifolius* and *R. crispus*, respectively). 

Analysis of the vertical distribution of proanthocyanidins in the plant at the flowering stage showed that the root parts of both sorrel species located closer to the stem contained a higher level of these compounds (Figure 3b). Almost homogenous distribution of proanthocyanidins was found over the entire height of the *R. crispus* plant stem, while for *R. obtusifolius*, the maxima were in the lower part (eighth internode) and the upper parts (from first to third internodes). 

In general, the content of proanthocyanidins in the leaves of *R. crispus* increased from the root leaves to the terminal ones. As for *R. obtusifolius*, we detected a relatively high level of this class of compounds in the root leaves. Interestingly, the concentration of proanthocyanidins sharply decreased at the second node and then gradually increased in the uppermost young leaves, reaching the maximum value of the contents. 

At the fruiting stage, the maximum content of proanthocyanidins in the roots of R. crispus was in the first part (1.93 ± 0.12 mg CyE g^−1^), while the remaining parts of the root contained about two times less proanthocyanidins (Figure 3c).

We did not note any difference in the content of proanthocyanidins between the two upper parts of the *R. obtusifolius* roots. However, the measured value was higher compared with that of the lower part. The content of proanthocyanidins had a tendency to increase towards upper parts of the of *R. obtusifolius* stem and *R. crispus* leaves. Also, we determined a high level of proanthocyanidins in the *R. obtusifolius* root leaves. It is worth noting that the root leaves of this type of sorrel contained the same high amount of compounds of this class as the terminal leaves and seeds. 

### 3.3. Total Phenolic Content

**Figure 4:**
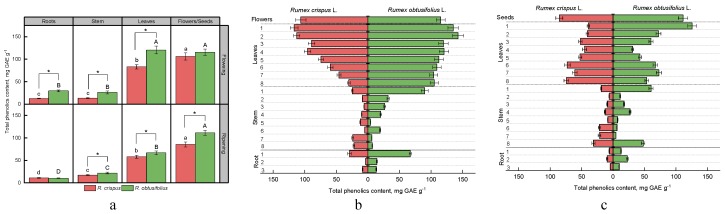
The content of phenolic compounds in different parts of plants *R. crispus* and *R. obtusifolius* at flowering and fruiting stages (**a**), and vertical distribution of phenolic compounds at flowering stage (**b**) and at fruiting stage (**c**). Different lower case letters indicate significant differences among plant parts of *R. crispus*; upper case letters indicate significant differences among plant parts of *R. obtusifolius* (*p* ≤ 0.05); asterisk * indicates significant differences among sorrel species (*p* ≤ 0.05) based on post hoc Tukey’s tests separately for each growth stage.

Phenolic compounds represent a class of secondary metabolites that are widely distributed across plant organisms, including several thousand compounds with different structures (from simple phenolic acids to complex polymer compounds, tannins). This study aims to investigate the total content of phenolic compounds in various plant organs and tissues of *R. crispus* and *R. obtusifolius* at flowering and fruiting stage. As for the flowering stage, the content of phenolic compounds was considerably higher in the roots, stem, and leaves of *R. obtusifolius,* while it was not significantly different in the flowers of both species (Figure 4a). 

At the fruiting stage, we determined a higher level of phenolic compounds in the stem, leaves, and seeds of *R. obtusifolius* compared with samples of *R. crispus*. The maximum difference between two sorrel varieties was found in the leaves at the flowering stage (83.2 ± 5.0 mg GAE g^−1^ and 120.5 ± 8.6 mg GAE g^−1^ for *R. crispus* and *R. obtusifolius*, respectively). On average, leaves and generative parts of sorrel plants accumulated 3–7 times higher levels of phenolic compounds compared with roots and stems. In contrast with the flowering stage, the total phenolic content decreased in all the studied parts of the sorrel with the onset of fruiting, except for the *R. crispus* stem. A particularly strong difference was observed in the leaves of *R. obtusifolius*, where the level phenolic compounds at the fruiting stage was two times lower compared with samples of leaves collected at the flowering stage.

Analysis of the vertical distribution of phenolic compounds in sorrel roots at the flowering stage showed that the content of compounds in question was highest in the upper part of the root (Figure 4b). Regarding the stem of *R. obtusifolius* and leaves of *R. crispus,* we observed a gradual increase in the content of phenolic compounds from the lower parts to the upper parts. The *R. crispus* stem indicated a relatively high level of phenolic compounds in the lower parts (in the eighth and seventh internodes) and in the upper internode. Almost all samples of the *R. obtusifolius* leaves contained a high content of phenolic compounds (from 104.3 to 120.6 mg GAE g^−1^ between the eighth and sixth nodes), reaching the highest levels in the upper young leaves of the first and second nodes (135.1–142.9 mg GAE g^−1^). 

The distribution of the phenolic compounds in a plant at the fruiting stage was somewhat different. (Figure 4c). Both species indicated a higher content in the second part of the roots. Phenolic compounds appeared to accumulate in the root internode of the *R. crispus* stem. Apart from the eighth internode, the level of phenolic compounds was also high in the upper part of the *R. obtusifolius* stem located closer to the inflorescence. 

The amount of total phenolic compounds in *R. crispus* leaves tended to decrease from the lower older leaves to the upper younger ones. In contrast, in *R. obtusifolius*, the maximum level of phenolic compounds was observed in the leaves of the first node.

### 3.4. Total Antioxidant Activity (AOA)

**Figure 5:**
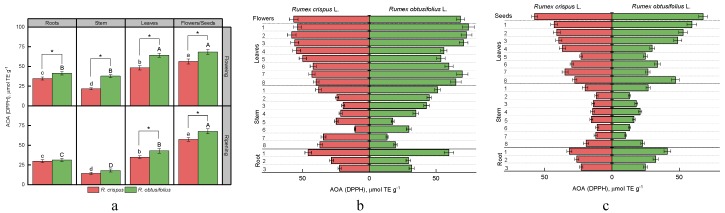
Antioxidant activity of extracts of various plant parts of *R. crispus* and *R. obtusifolius* (according to the 1,1-diphenyl-2-picrylhydrazyl (DPPH) method) at the flowering and fruiting stages (**a**), antioxidant activity according for vertical distribution at flowering stage (**b**) and at fruiting stage (**c**). Different lower case letters indicate significant differences among plant parts of *R. crispus*; upper case letters indicate significant differences among plant parts of *R. obtusifolius* (*p* ≤ 0.05); asterisk * indicates significant differences among sorrel species (*p* ≤ 0.05) based on post hoc Tukey’s tests separately for each growth stage.

**Figure 6:**
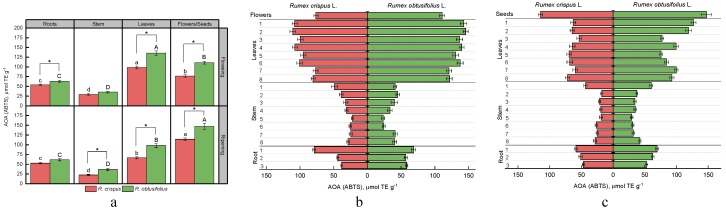
Antioxidant activity of extracts of various plant parts of *R. crispus* and *R. obtusifolius* (according to the 2,2’azinobis(3)ethylbenzthiazoline-6-sulfonic acid (ABTS) method) at the flowering and fruiting stages (**a**), antioxidant activity according for vertical distribution at flowering stage (**b**) and at fruiting stage (**c**). Different lower case letters indicate significant differences among plant parts of *R. crispus*; upper case letters indicate significant differences among plant parts of *R. obtusifolius* (*p* ≤ 0.05); asterisk * indicates significant differences among sorrel species (*p* ≤ 0.05) based on post hoc Tukey’s tests separately for each growth stage.

**Figure 7:**
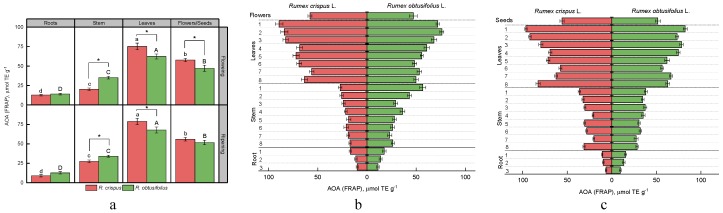
Antioxidant activity of extracts of various plant parts of *R. crispus* and *R. obtusifolius* (according to the ferric reducing antioxidant power (FRAP) method) at the flowering and fruiting stages (**a**), antioxidant activity according for vertical distribution at flowering stage (**b**) and at fruiting stage (**c**). Different lower case letters indicate significant differences among plant parts of *R. crispus*; upper case letters indicate significant differences among plant parts of *R. obtusifolius* (*p* ≤ 0.05); asterisk * indicates significant differences among sorrel species (*p* ≤ 0.05) based on post hoc Tukey’s tests separately for each growth stage.

To date, there are a variety of methods for studying the antioxidant activity of plant materials. DPPH and ABTS methods are widely used to evaluate the antioxidant activity of food products. They are based on the ability of antioxidants to reduce the DPPH and ABTS radicals to non-radical forms. FRAP analysis is a further method of assessing electron donating activity, which is considered to be an essential mechanism of antioxidant activity. This method serves as an indicator of the reducing power of plant extracts. To compare the data obtained by all three methods, we used Trolox as a standard in this work. Figure 5, Figure 6 and Figure 7 present the results of the antioxidant activity of sorrel extracts. 

The antioxidant activity of extracts from different parts of two sorrel varieties, determined by the method of DPPH and ABTS, differed in absolute values by about two times, but had a similar correlation (Figure 5a and Figure 6a, Table 2). On average, plants of *R. obtusifolius* revealed higher antioxidant activity. We were able to rank the observed parts of sorrel plants in descending order of antioxidant activity (according to the DPPH method) as follows: the generative part (flowers, seeds) > leaves > root > stem (for flowering and fruiting stages). The ABTS method showed the same results for both species at the stage of seed ripening; however, at the flowering stage, the antioxidant activity of leaf extracts was higher compared with the generative part. Analysis of the antioxidant activity of various sorrel parts in the vertical projection of a plant using the methods of DPPH and ABTS revealed that parts of the root closer to the stem tended to have higher activity (Figure 5b and Figure 6b). We also observed a high antioxidant activity of extracts made from the stem internodes positioned close to the roots and those adjacent to the generative part of internodes. The exceptions were the samples of the stem of *R. obtusifolius* collected at the flowering stage. In this case, there was a tendency to increase antioxidants from the lower internodes to the upper ones. For analysis of *R. crispus* leaves collected at the flowering and fruiting stages, the antioxidant activity (DPPH) increased from the root leaves towards the terminal ones (Figure 5b,c). The antioxidant activity (DPPH) of the leaves of *R. obtusifolius* was high in the root zones, after which it decreased and subsequently increased in the leaves of the upper nodes. The antioxidant activity of leaf extracts measured by ABTS assay demonstrated somewhat different results. For samples of *R. crispus* at the fruiting stage and *R. obtusifolius* at the flowering stage, the level of the antioxidant activity practically did not depend on the vertical position of the leaves. As for the rest of the samples, a general pattern was found regarding an increase in antioxidant activity from the lower sections to the upper ones (Figure 6b,c).

The results obtained from the FRAP analysis of the antioxidant activity in different parts of the two sorrel plant varieties were not quite comparable to those of the methods of DPPH and ABTS (Figure 7). A higher level of antioxidant activity was found in samples of the stem and roots of *R. obtusifolius,* while the generative part and leaves of this sorrel type were characterized by less antioxidant activity than the same parts of *R. crispus* (Figure 7a). According to the FRAP method, the sorrel parts studied can be ranked in descending order of antioxidant activity as follows: leaves > the generative part (flowers, seeds) > stem > root (at flowering and fruiting stages). The analysis of the vertical distribution of antioxidants by the FRAP method revealed, in general, a clear trend of gradual increase in the antioxidant activity from the lowest parts of the root to the upper leaves (Figure 7b,c). However, the terminal leaves of *R. crispus* showed, at the fruiting stage, an antioxidant activity comparable to that of younger leaves of the first node (Figure 7c). The generative part of both species, at the stage of both flowering and fruiting, appeared to have a lower antioxidant activity than leaves. 

## 4. Discussion

Our study investigated the accumulation of phenolic compounds and the antioxidant activity of extracts of various parts of *R. crispus* and *R. obtusifolius*, collected at the flowering and fruiting stages. Earlier, several studies showed that this period of ontogenesis is characterized by the maximum content of phenolic compounds in the aerial part of *Rumex crispus* plants [47,48]. It should be noted that many herbaceous plants are reported to have peak levels of secondary metabolites, including phenolic compounds, in these phenological phases [49,50,51]. The changes observed may arise from an increase in the activity of phenylalanilammiase lyase (PAL). In the work of Andreotti et al., it was shown that the concentration of this enzyme reached its maximum during the initiation of flowering [52].

The stem part of two sorrel varieties indicated the lowest values of the parameters studied. The antioxidant and phenolic properties can be attributed to two factors: the intrinsic metabolic activity of the cells and tissues of the stem and the molecular composition of the exudate transported through the phloem channels. It should be noted that phenolic compounds are not, apparently, typical compounds for phloem transport. However, many authors indicate the presence of a small number of phenolic substances in phloem exudates [53]. One of the factors preventing the free flow of phenols through phloem channels is the alkaline reaction of the central cavity solution, in which these compounds are particularly sensitive to oxidation, forming aggressive quinones. 

Therefore, it is more likely that the synthesis of phenolic compounds, oligomerization, and “decoration” (glycosylation, methylation) occurs in the cells accumulating these products, rather than being transported to them from the leaf in ready-made form. Thus, photosynthesizing mesophilic tissue or the metabolism of a typical phloem exudate, sucrose, makes the main contribution to the formation of the phenol profile of the stem part of the sorrel species studied. 

At the same time, this does not exclude the possibility of transporting small amounts of polyphenols, which probably play a specific regulatory role, through the conducting systems of the stem (link). Thus, quinic and shikimic acid found in the phloem exudate can be considered as precursors of aromatic rings of phenolic compounds. Also, these compounds contribute to the evaluation of indicators such as total antioxidant activity (DPPH, ABTS, FRAP), as well as to the evaluation of the TPC parameter. 

A study conducted on *Cistus clusii* shows that from a certain age, the "pressure" of oxidative stress in chloroplasts gradually increases, while the photosynthetic activity of mesophyll cells decreases [54]. The leaves of the root rosette are the most aged, while the leaves of the upper internodes (adjacent to inflorescences or fruits) appear at the later stages of ontogenesis. The results of the DPPH, ABTS, and FRAP tests show that the antioxidant status of the aging plant organs is reduced compared with that of the young ones. At the same time, the biosynthesis of secondary metabolites, directly related to the productivity of the photosynthetic apparatus, slows down in the leaves of the root rosette and lower internodes. A similar trend has been demonstrated in *Ilex paraguariensis* plants. Thus, leaves that reached six months were characterized by low TPC values, compared with leaves of one and two months of age [55]. Although the data regarding the influence of the age of the lamina on metabolic productivity are somewhat contradictory, most authors are inclined to the hypothesize that young tissues usually have higher rates of metabolism biosynthesis [56,57,58,59]. 

The biosynthesis of secondary metabolites in the rhizome and roots of *R. crispus* and *R. obtusifolius* is mostly associated with the transport of photoassimilates through the plant’s conducting systems. The most characteristic components of phloem exudate, as mentioned earlier, are carbohydrates, which act like a characteristic precursor for the biosynthesis of phenolic metabolites. However, some data indicate the presence of amino acids, including the aromatic series, in the phloem exudate [60,61]. Thus, phenylalanine and tyrosine can be directly involved in the biosynthesis of secondary compounds of phenolic nature. Besides, the experiments on woody plants have shown that introduction of exogenous quinic acid, which is quickly involved in the downward transport, contributes to further lignification of cambial structures [62]. As the underground parts of sorrel plants tend to stiffen while forming a rhizome, the potential transport of phenolic acids can play a significant role in the lignification of this structure, thus making a significant contribution to the phenolic status of the underground parts of plants [63].

## 5. Conclusions

Our work demonstrated that the sorrel species studied have a similar tendency to the accumulation of biologically active substances, which is partly determined by the homogeneity of the place of growth and similar climatic conditions. Also, it is worth noting the high correlation between the level of the fractions of phenolic compounds in question and the antioxidant activity of the extracts. As plant polyphenols show a common tendency to inhibit free radical reactions in vitro and in vivo owing to their redox properties, the plant samples tested can play an essential role as biotechnological objects as well as valuable sources of phenolic compounds and flavonoids. 

## Figures and Tables

**Table 1:** Results of three-factorial analysis of variance (ANOVA) for phenolic compounds and antioxidant activity of sorrel extracts.

Factor	Factor Level	TCC	PAs	TPC	AOA (DPPH)	AOA (ABTS)	AOA (FRAP)
Main effects							
Species (Sp)	*R. crispus*	3.12 b	7.5 b	48.4 b	37.2 b	64.3 b	42.1 a
	*R. obtusifolius*	3.43 a	10.7 a	62.7 a	46.4 a	86.0 a	40.7 a
Plant part (P)	Roots	1.93 c	5.5 c	15.9 c	34.3 c	58.0 c	12.1 d
	Stem	1.09 d	3.9 c	19.8 c	22.9 d	30.8 d	29.2 c
	Leaves	4.00 b	10.4 b	82.1 b	47.5 b	99.6 b	71.1 a
	Flowers/Seeds	5.95 a	16.6 a	104.6 a	62.5 a	112.2 a	53.2 b
Growth stage (GS)	Flowering	3.03 b	8.6 a	63.4 a	46.6 a	75.4 a	40.6 a
	Ripening	3.45 a	9.6 a	47.7 b	37.0 b	74.9 a	42.2 a
Significance							
	Sp	0.046 *	<0.001 *	<0.001 *	0.024 *	<0.001 *	0.086 ^ns^
	P	<0.001 *	0.028 *	0.035 *	<0.001 *	<0.001 *	<0.001 *
	GS	<0.001 *	0.112 ^ns^	<0.001 *	<0.001 *	0.142 ^ns^	0.054 ^ns^
	Sp*P	<0.001 *	<0.001 *	<0.001 *	<0.001 *	<0.001 *	<0.001 *
	Sp*GS	<0.001 *	<0.001 *	0.008 *	<0.001 *	<0.001 *	0.032 *
	P*GS	<0.001 *	0.263 ^ns^	<0.001 *	<0.001 *	<0.001 *	<0.001 *
	Sp*P*GS	<0.001 *	<0.001 *	0.018 *	0.020 *	<0.001 *	0.046 *

Data were evaluated via three-way ANOVA, factors: species, plant part, and growth stage, followed by Tukey HSD (honestly significant difference) test (mean, *n* = 4). Identical letters indicate that values do not differ significantly. Asterisks (*) indicate significantly influential factors. ns, not significant; TCC, total catechins content; PA, proanthocyanidins; TPC, total phenolic content; AOA, total antioxidant activity; DPPH, 1,1-diphenyl-2-picrylhydrazyl; ABTS^+^, 2,2’azinobis(3)ethylbenzthiazoline-6-sulfonic acid; FRAP, ferric reducing antioxidant power.

**Table 2:** Correlation matrix with the Pearson coefficient values for the phenolic compounds and antioxidant activity of sorrel extracts.

Parameters	TCC	PAs	TPC	AOA (DPPH)	AOA (ABTS)	AOA (FRAP)
TCC	1.0000					
PAs	0.6020 (*p* < 0.0001)	1.0000				
TPC	0.6343 (*p* < 0.0001)	0.4894 (*p* < 0.0001)	1.0000			
AOA (DPPH)	0.6255 (*p* < 0.0001)	0.4396 (*p* < 0.0001)	0.8789 (*p* < 0.0001)	1.0000		
AOA (ABTS)	0.6859 (*p* < 0.0001)	0.3867 (*p* < 0.0001)	0.8741 (*p* < 0.0001)	0.8125 (*p* < 0.0001)	1.0000	
AOA (FRAP)	0.6803 (*p* < 0.0001)	0.6701 (*p* < 0.0001)	0.7032 (*p* < 0.0001)	0.5523 (*p* < 0.0001)	0.6381 (*p* < 0.0001)	1.0000
